# Correction: Dietary nitrate supplementation prevents radiotherapy-induced xerostomia

**DOI:** 10.7554/eLife.107890

**Published:** 2025-06-04

**Authors:** Xiaoyu Feng, Zhifang Wu, Junji Xu, Yipu Xu, Bin Zhao, Baoxing Pang, Xingmin Qu, Liang Hu, Lei Hu, Zhipeng Fan, Luyuan Jin, Dengsheng Xia, Shimin Chang, Jingsong Wang, Chunmei Zhang, Songlin Wang

**Keywords:** Other

Feng X, Wu Z, Xu J, Xu Y, Zhao B, Pang B, Qu X, Hu L, Hu L, Fan Z, Jin L, Xia D, Chang S, Wang J, Zhang C, Wang S. 2021. Dietary nitrate supplementation prevents radiotherapy-induced xerostomia. *eLife* **10**:e70710. doi: 10.7554/eLife.70710.

Published 28 September 2021

Going through our publication again, we found some inadvertent duplications in Figure 4, Figure 5, and Figure 4—figure supplement 1. These errors occurred during figure assembly, and do not affect the interpretation and integrity of this work. No changes to the main text and figure legends were made relating to these errors. We sincerely apologize for the oversight.

In Figure 4h the images of Scramble 72 h, siRNA-2 24 h and siRNA-2 48 h duplicate or overlap with the images of Scramble 48 h, siRNA-1 48 h and siRNA-1 72 h, respectively. We captured a large number of EdU +fluorescent images and also different microscopic views obtained from the same well. The errors in Figure 4h were introduced during figure assembly. The raw images for Figure 4h were stored as adjacent files, and due to erroneous selection of representative images this resulted in panels being duplicated or with overlapping views. These errors have been corrected by replacing the originally published images of Scramble 72 h, siRNA-2 24 h and siRNA-2 48 h in Figure 4h.

The IR groups are duplicated between Figure 5c, d and Figure 4c, d, respectively, and Sham groups duplicated between Figure 5c and Figure 4—figure supplement 1b. These duplications occurred during figure assembly, where we use a shared template but forgot to delete/replace the templates with the correct data for each IR and Sham group. We’d like to clarify that the duplication of IR and Sham groups exhibit the results of the same cell under the identical experimental conditions. We have replaced the duplicated images of Figure 5c, d and Figure 4—figure supplement 1b in the corrected version.

Additionally, in Figure 4l and Figure 4—figure supplement 1b we inadvertently selected the wrong representative images for WT 48 h and Nitrate 24 h, respectively. This error occurred as several of the raw mages from a parallel study were stored not only in their designated folders but were erroneously saved into Figure 4l (WT 48 h) and Figure 4—figure supplement 1b (Nitrate 24 h). These images displayed comparable experimental trends to the proper WT 48 h and Nitrate 24 h datasets, so we did not notice that initially. We have replaced these panels in Figure 4l and Figure 4—figure supplement 1b with the correct images. Lastly, the scale bars were missing in Figure 5i and have been added in the corrected version.

The corrected version of Figure 4 is shown here:

**Figure 1:**
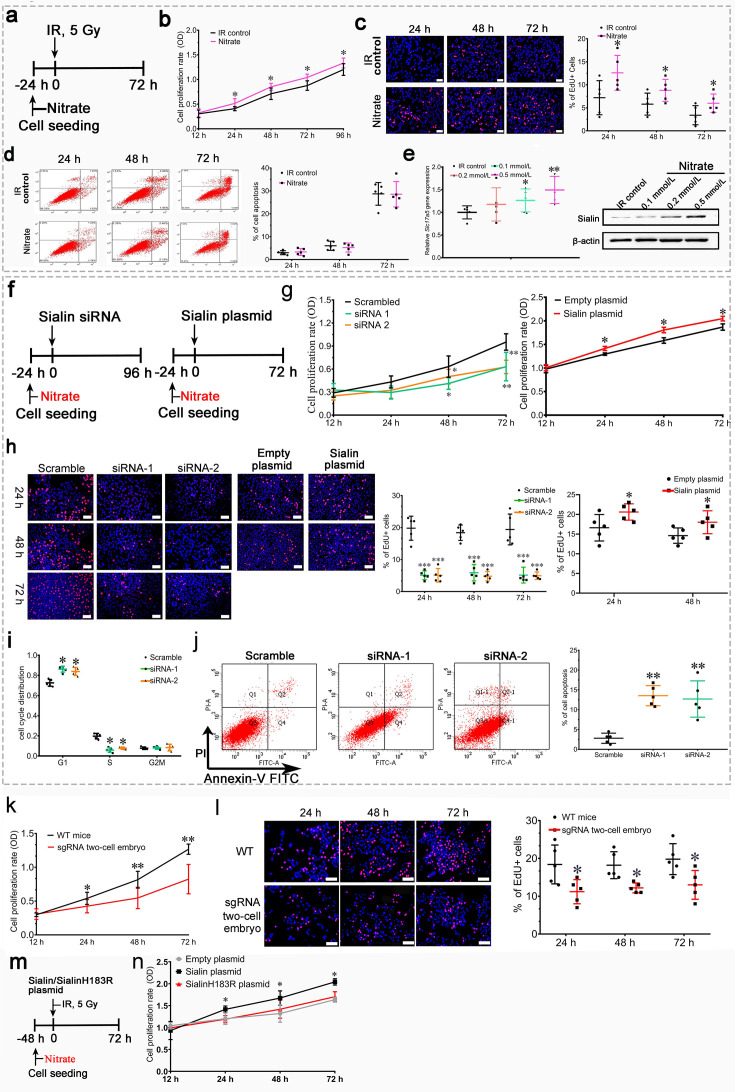


The originally published Figure 4 is shown for reference:

**Figure 4:**
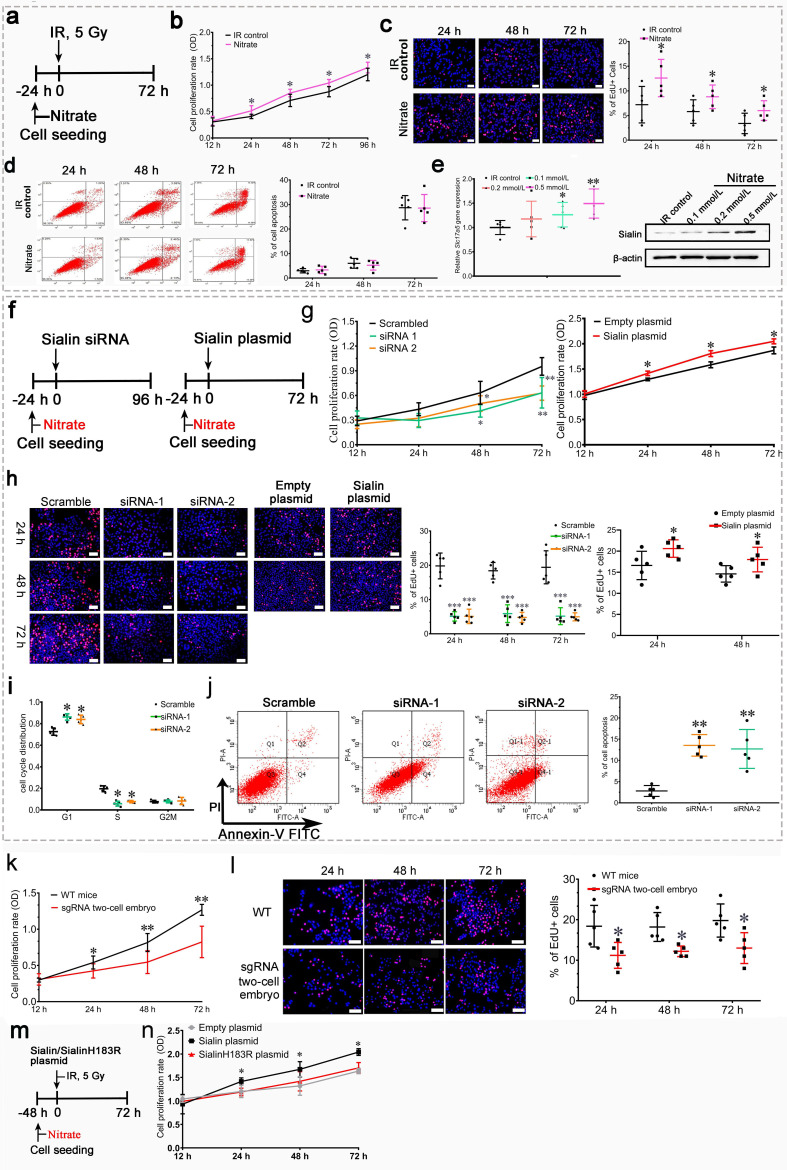


The corrected version of Figure 5 is shown here:

**Figure 2:**
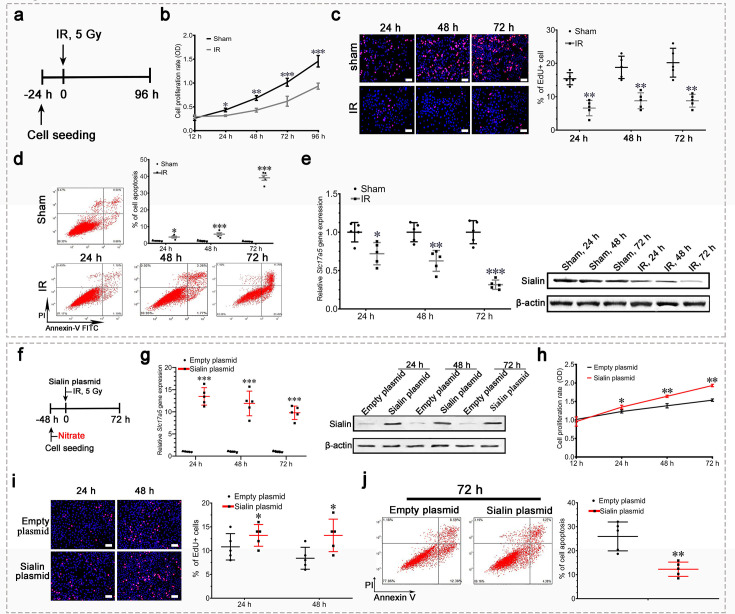


The originally published Figure 5 is shown for reference:

**Figure 5:**
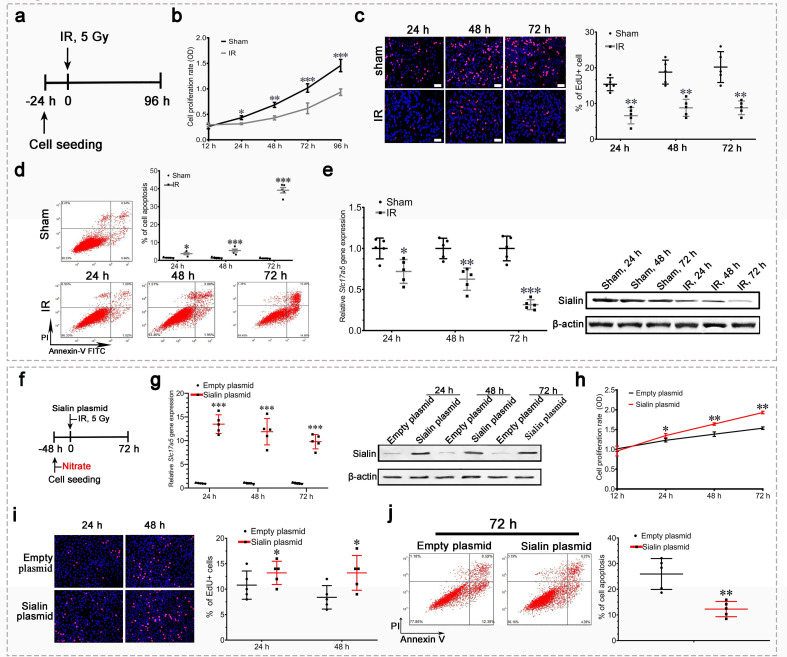


The corrected version of Figure 4—figure supplement 1 is shown here:

**Figure 3:**
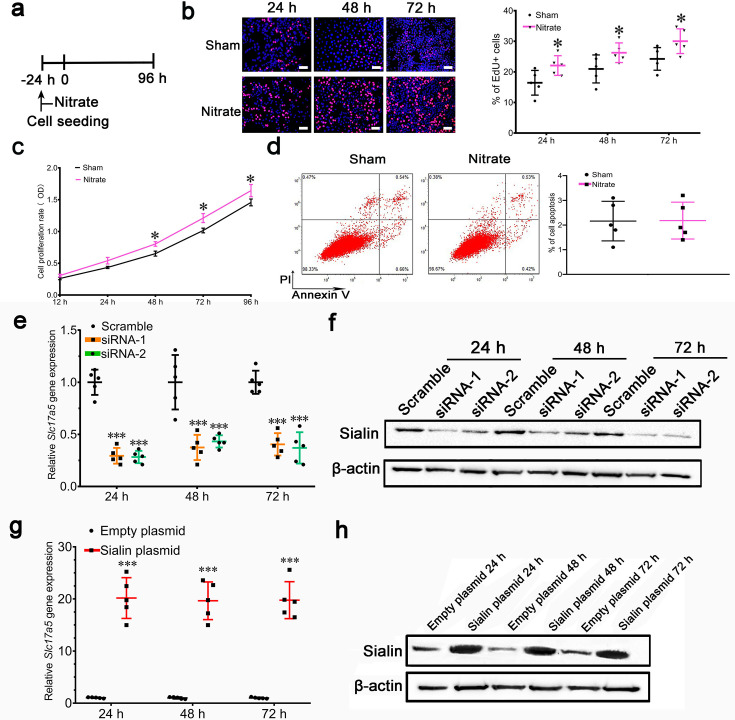


The originally published Figure 4-figure supplement 1 is shown for reference:

**Figure 6:**
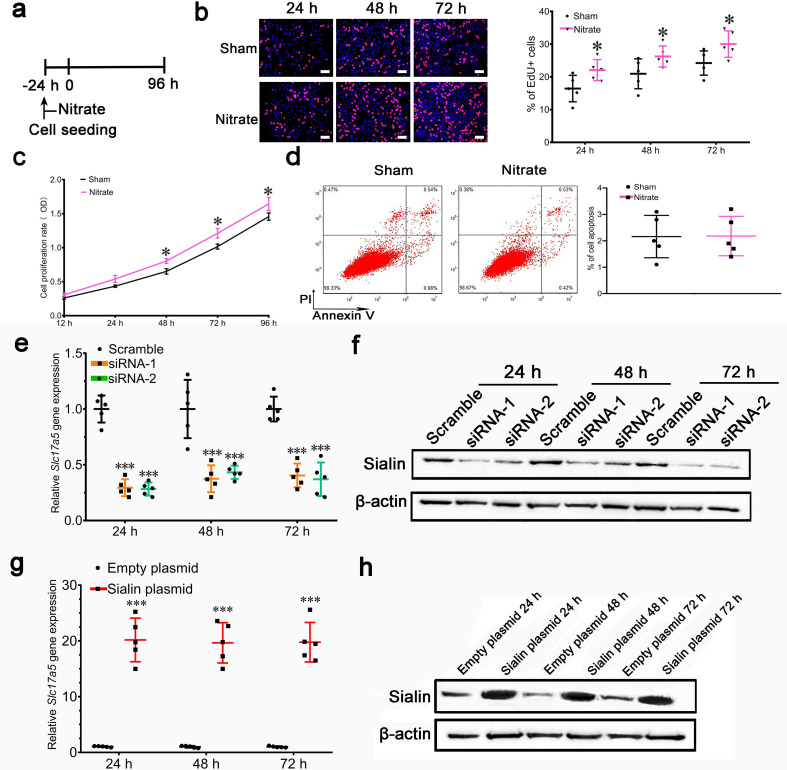


The article has been corrected accordingly.

