# Supplementary figures and images for: Dietary nitrate supplementation prevents radiotherapy-induced xerostomia
Source: eLife. 2021 Sep 28;10:e70710. doi: 10.7554/eLife.70710 (PMC8563005; doi:10.7554/eLife.70710)

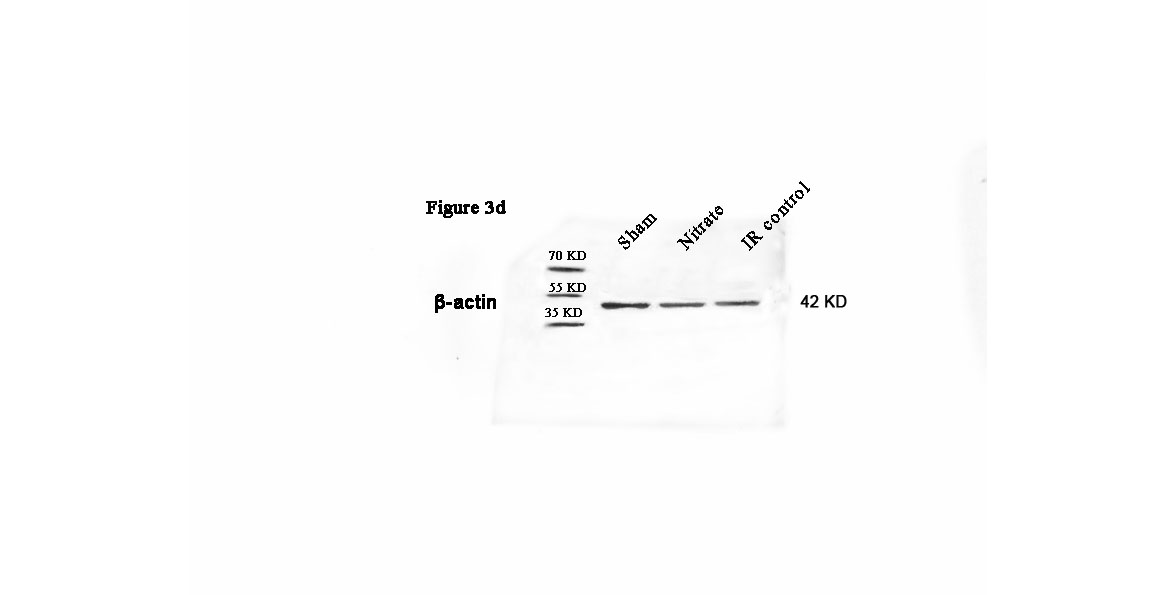

Supplement: Figure 3—source data 1. [file elife-70710-fig3-data1.zip › Figure 3 source data1/Figure 3d actin.jpg]

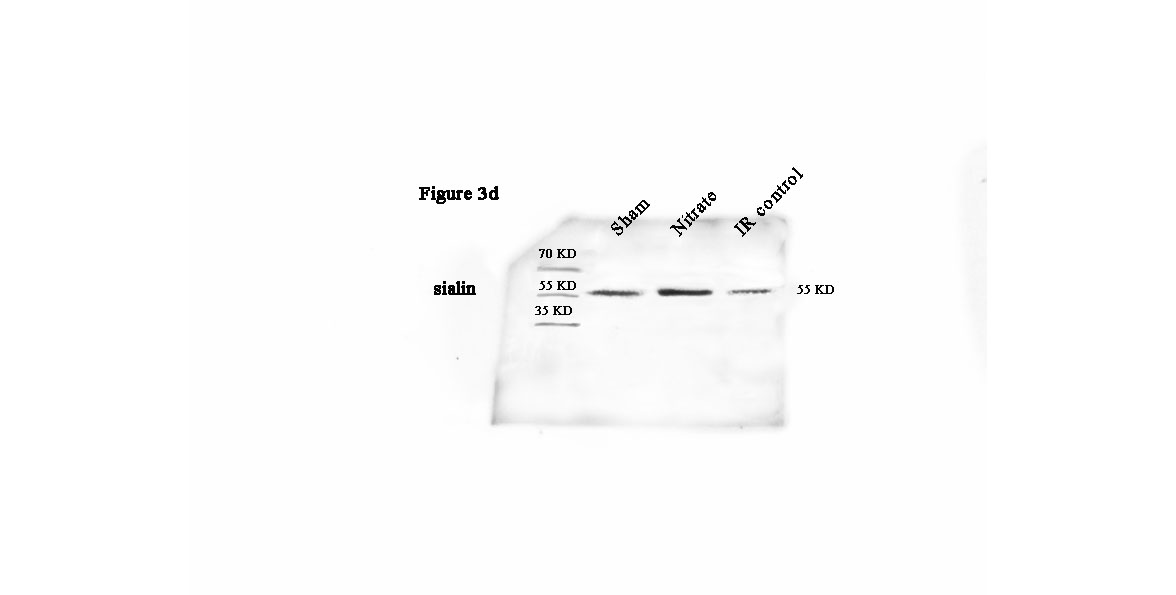

Supplement: Figure 3—source data 1. [file elife-70710-fig3-data1.zip › Figure 3 source data1/Figure 3d sialin.jpg]

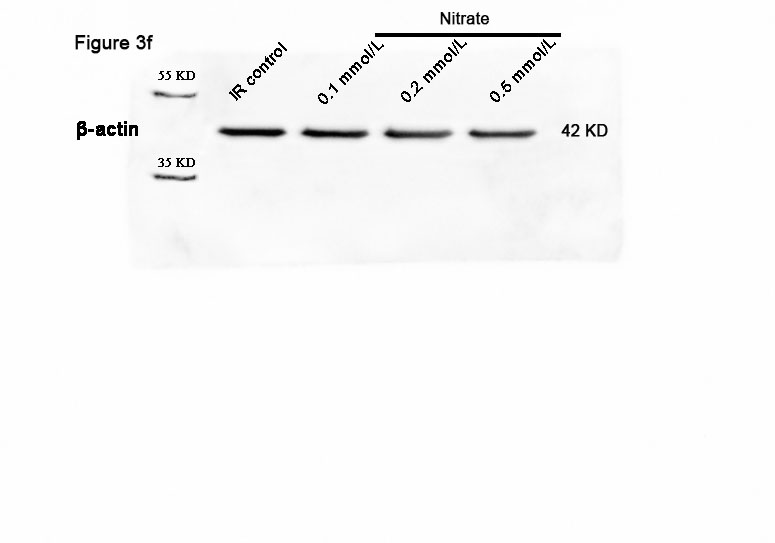

Supplement: Figure 3—source data 2. [file elife-70710-fig3-data2.zip › Figure 3 source data2/Figure 3f actin.jpg]

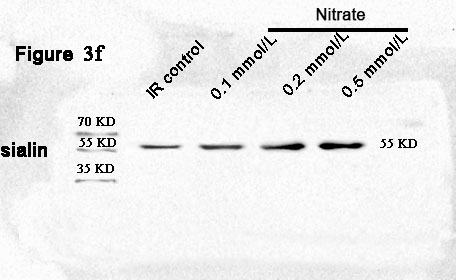

Supplement: Figure 3—source data 2. [file elife-70710-fig3-data2.zip › Figure 3 source data2/Figure 3f sialin.jpg]

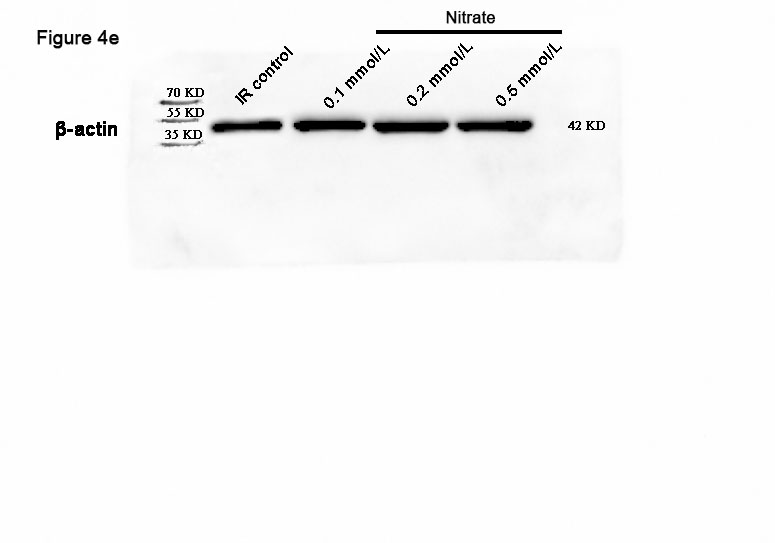

Supplement: Figure 4—source data 1. [file elife-70710-fig4-data1.zip › Figure 4 source data1/Figure 4e actin.jpg]

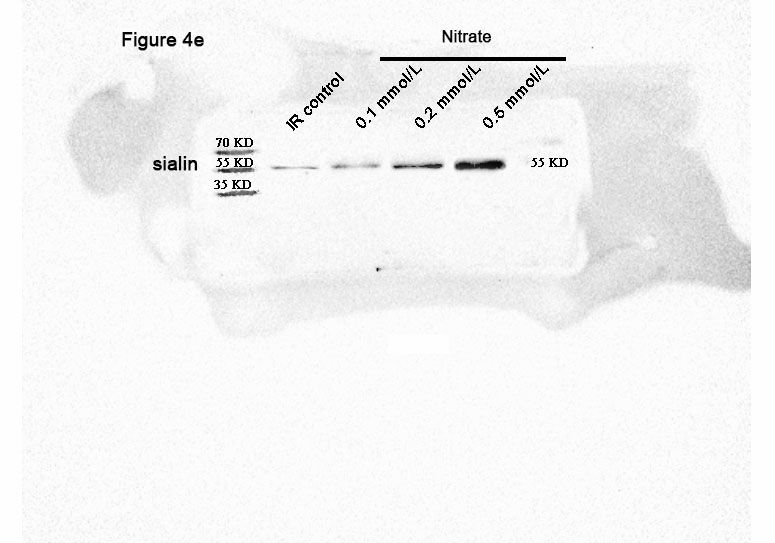

Supplement: Figure 4—source data 1. [file elife-70710-fig4-data1.zip › Figure 4 source data1/Figure 4e sialin.jpg]

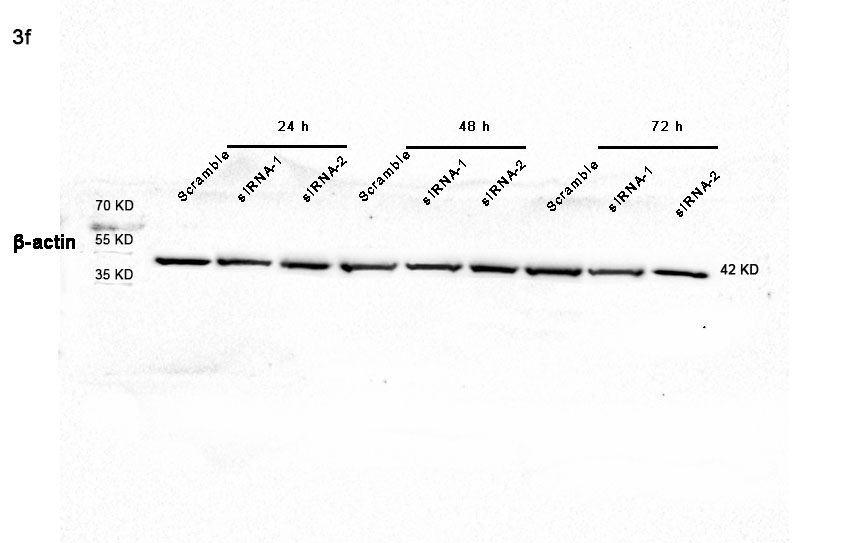

Supplement: Figure 4—figure supplement 1—source data 1. [file elife-70710-fig4-figsupp1-data1.zip › Figure 4-figure supplement 3 source data1/3f actin.jpg]

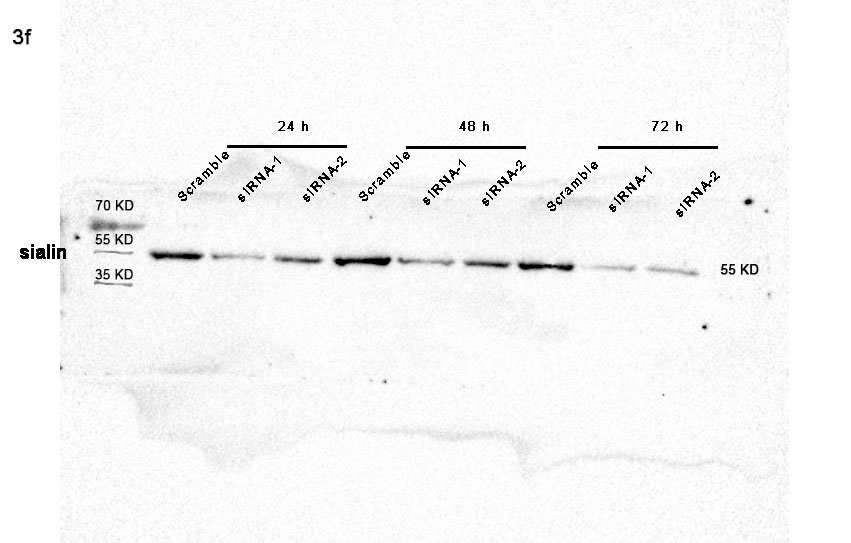

Supplement: Figure 4—figure supplement 1—source data 1. [file elife-70710-fig4-figsupp1-data1.zip › Figure 4-figure supplement 3 source data1/3f sialin.jpg]

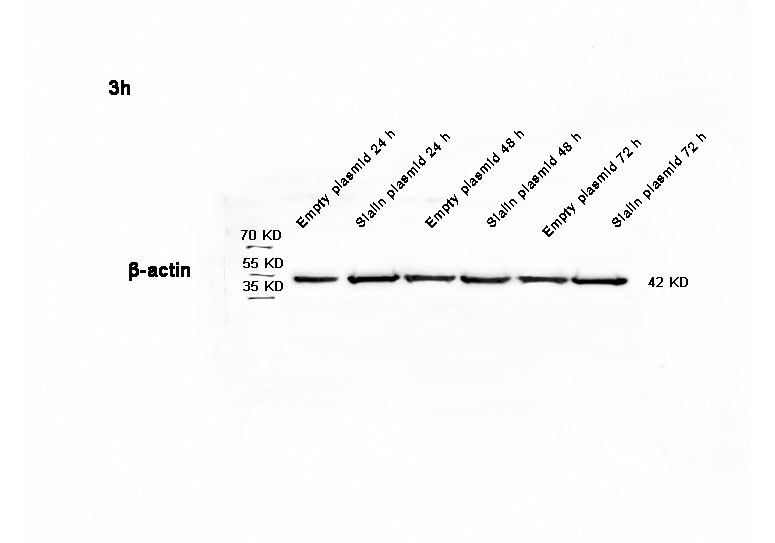

Supplement: Figure 4—figure supplement 1—source data 2. [file elife-70710-fig4-figsupp1-data2.zip › Figure 4-figure supplement 3 source data2/3h actin.jpg]

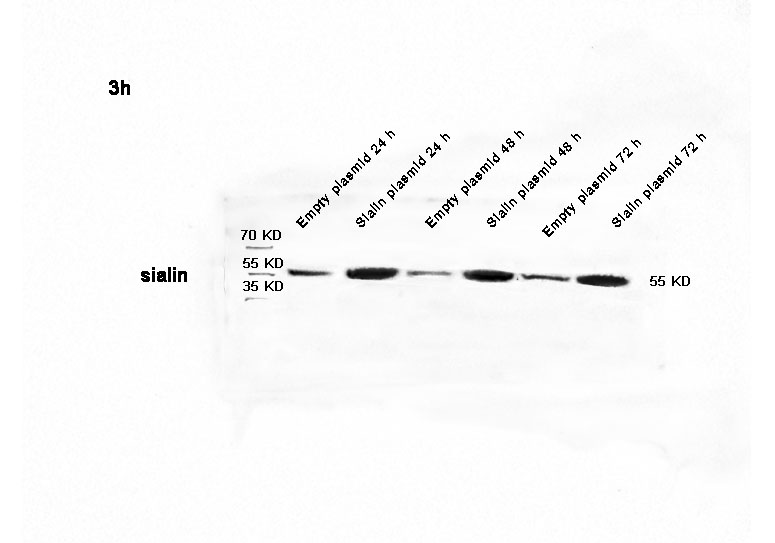

Supplement: Figure 4—figure supplement 1—source data 2. [file elife-70710-fig4-figsupp1-data2.zip › Figure 4-figure supplement 3 source data2/3h sialin.jpg]

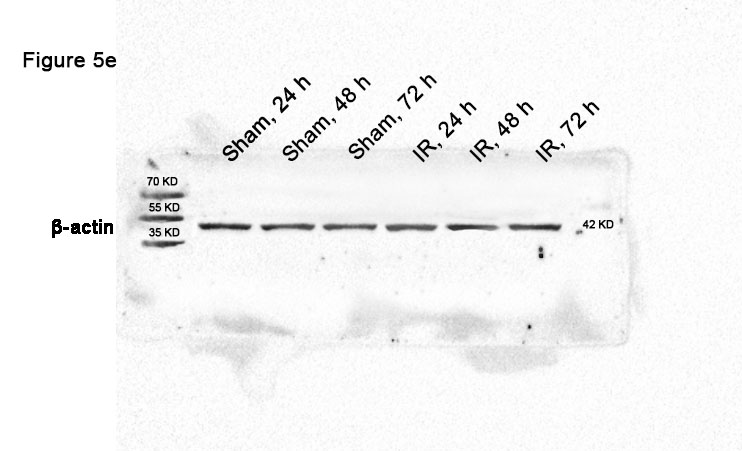

Supplement: Figure 5—source data 1. [file elife-70710-fig5-data1.zip › Figure 5 source data1/Figure 5e actin.jpg]

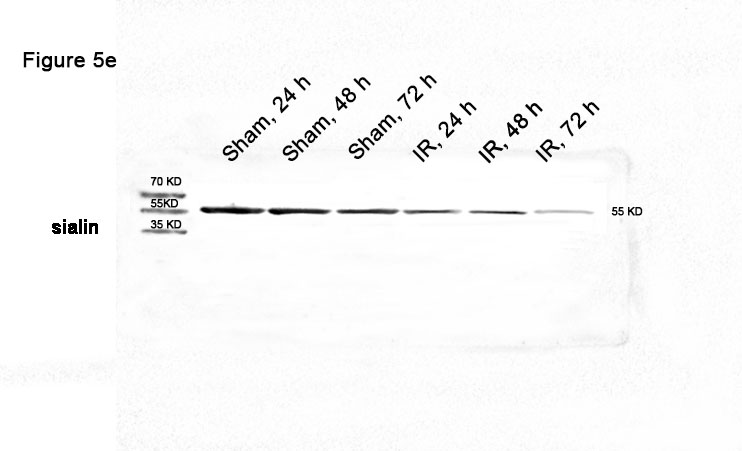

Supplement: Figure 5—source data 1. [file elife-70710-fig5-data1.zip › Figure 5 source data1/Figure 5e sialin.jpg]

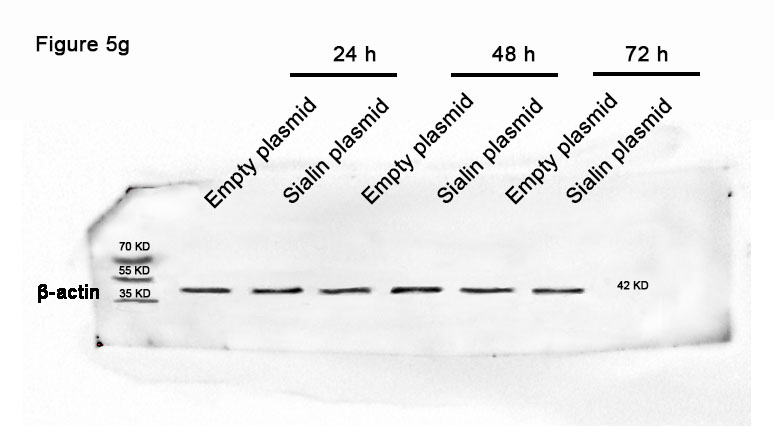

Supplement: Figure 5—source data 2. [file elife-70710-fig5-data2.zip › Figure 5 source data2/Figure 5g actin.jpg]

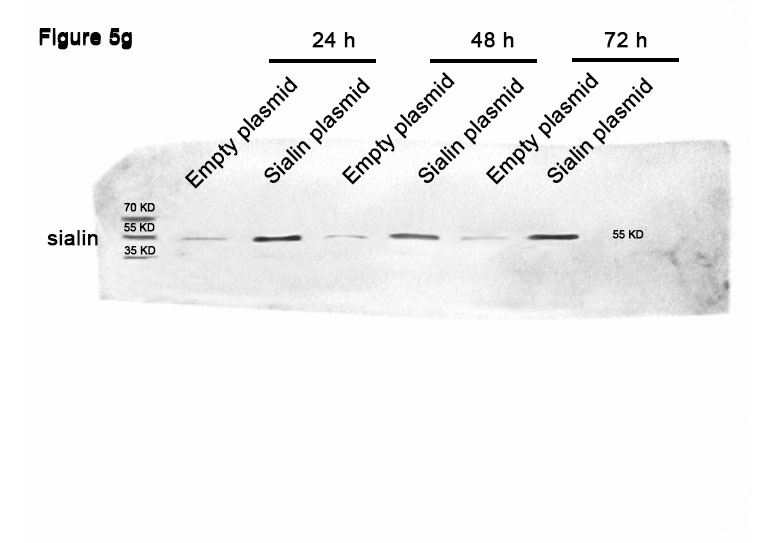

Supplement: Figure 5—source data 2. [file elife-70710-fig5-data2.zip › Figure 5 source data2/Figure 5g sialin.jpg]

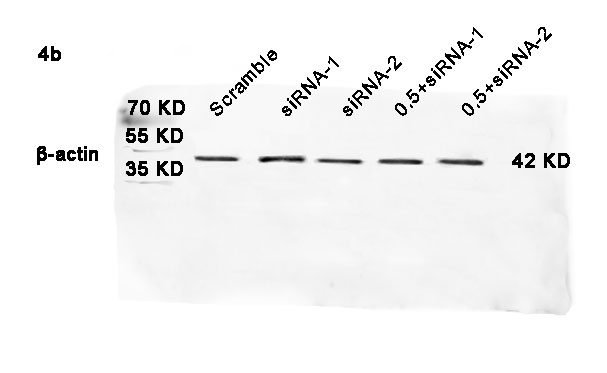

Supplement: Figure 5—figure supplement 1—source data 1. [file elife-70710-fig5-figsupp1-data1.zip › Figure 5-figure supplement 4 source data1/4b actin.jpg]

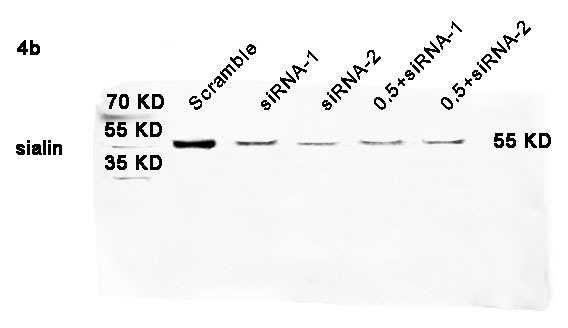

Supplement: Figure 5—figure supplement 1—source data 1. [file elife-70710-fig5-figsupp1-data1.zip › Figure 5-figure supplement 4 source data1/4b sialin.jpg]

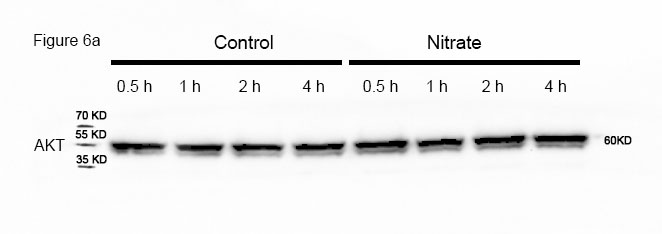

Supplement: Figure 6—source data 1. [file elife-70710-fig6-data1.zip › Figure 6 source data1/Figure 6a AKT.jpg]

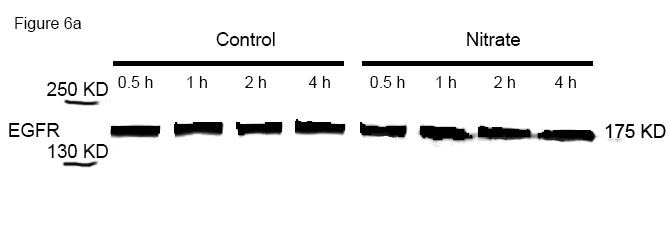

Supplement: Figure 6—source data 1. [file elife-70710-fig6-data1.zip › Figure 6 source data1/Figure 6a EGFR.jpg]

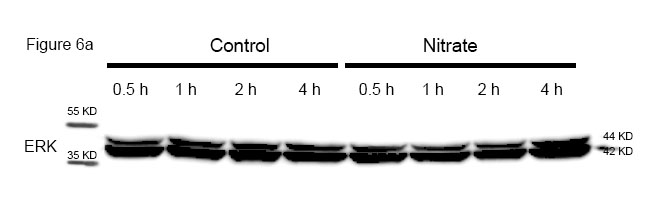

Supplement: Figure 6—source data 1. [file elife-70710-fig6-data1.zip › Figure 6 source data1/Figure 6a ERK.jpg]

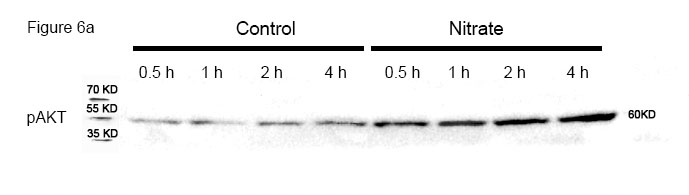

Supplement: Figure 6—source data 1. [file elife-70710-fig6-data1.zip › Figure 6 source data1/Figure 6a pAKT.jpg]

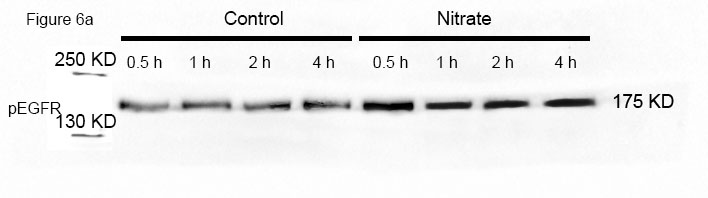

Supplement: Figure 6—source data 1. [file elife-70710-fig6-data1.zip › Figure 6 source data1/Figure 6a pEGFR.jpg]

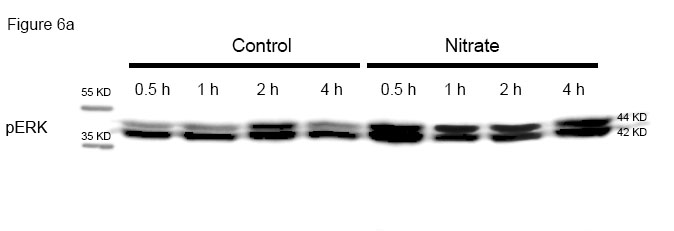

Supplement: Figure 6—source data 1. [file elife-70710-fig6-data1.zip › Figure 6 source data1/Figure 6a pERK.jpg]

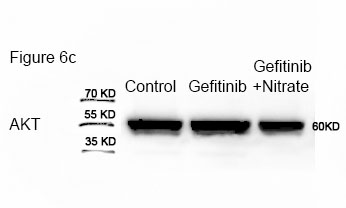

Supplement: Figure 6—source data 2. [file elife-70710-fig6-data2.zip › Figure 6 source data2/Figure 6c AKT.jpg]

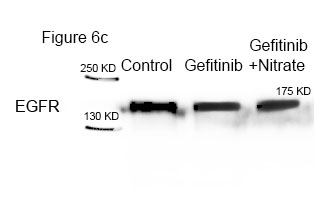

Supplement: Figure 6—source data 2. [file elife-70710-fig6-data2.zip › Figure 6 source data2/Figure 6c EGFR.jpg]

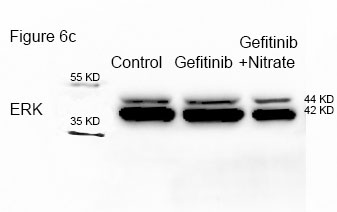

Supplement: Figure 6—source data 2. [file elife-70710-fig6-data2.zip › Figure 6 source data2/Figure 6c ERK.jpg]

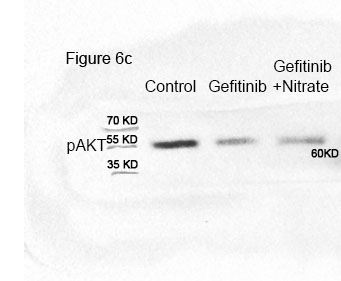

Supplement: Figure 6—source data 2. [file elife-70710-fig6-data2.zip › Figure 6 source data2/Figure 6c pAKT.jpg]

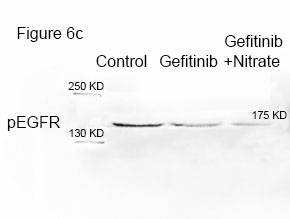

Supplement: Figure 6—source data 2. [file elife-70710-fig6-data2.zip › Figure 6 source data2/Figure 6c pEGFR.jpg]

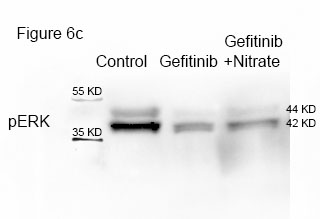

Supplement: Figure 6—source data 2. [file elife-70710-fig6-data2.zip › Figure 6 source data2/Figure 6c pERK.jpg]

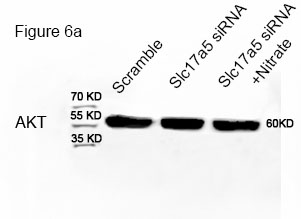

Supplement: Figure 6—source data 3. [file elife-70710-fig6-data3.zip › Figure 6 source data3/Figure 6a AKT slc17a5.jpg]

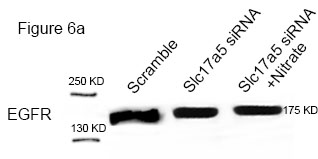

Supplement: Figure 6—source data 3. [file elife-70710-fig6-data3.zip › Figure 6 source data3/Figure 6a EGFR slc17a5.jpg]

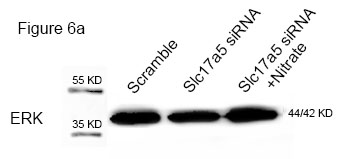

Supplement: Figure 6—source data 3. [file elife-70710-fig6-data3.zip › Figure 6 source data3/Figure 6a ERK slc17a5.jpg]

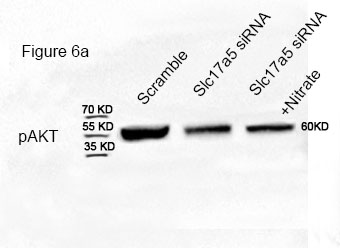

Supplement: Figure 6—source data 3. [file elife-70710-fig6-data3.zip › Figure 6 source data3/Figure 6a pAKT slc17a5.jpg]

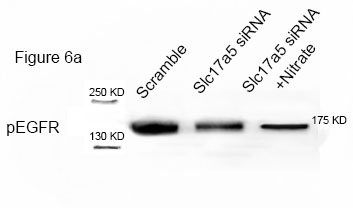

Supplement: Figure 6—source data 3. [file elife-70710-fig6-data3.zip › Figure 6 source data3/Figure 6a pEGFR slc17a5.jpg]

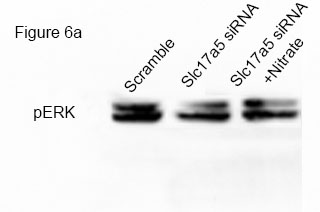

Supplement: Figure 6—source data 3. [file elife-70710-fig6-data3.zip › Figure 6 source data3/Figure 6a pERK slc17a5.jpg]

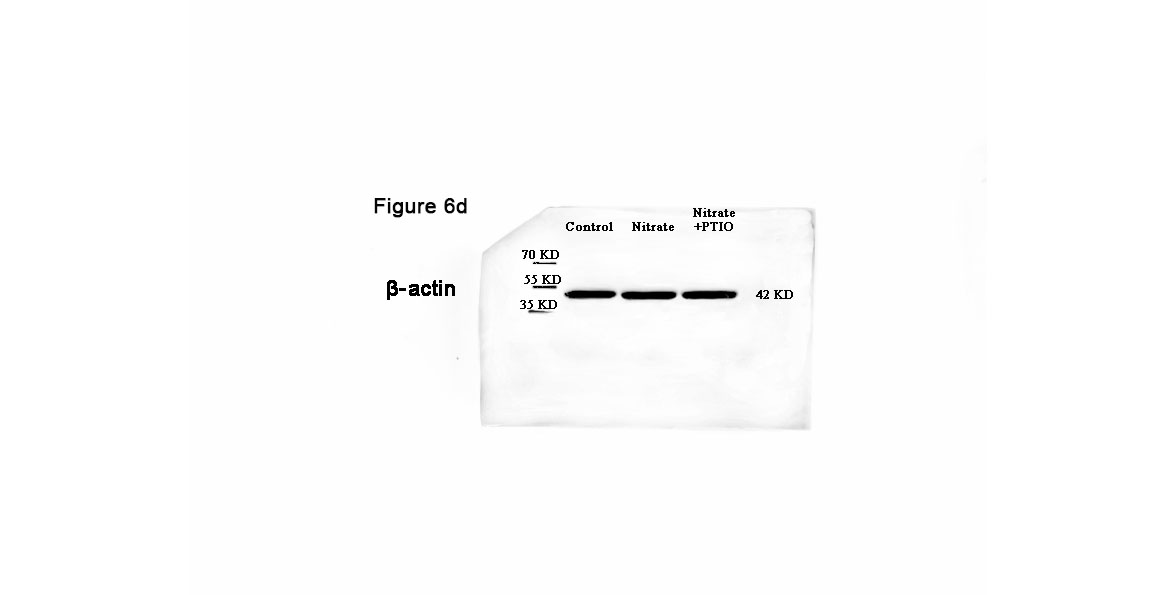

Supplement: Figure 6—source data 4. [file elife-70710-fig6-data4.zip › Figure 6 source data4/Figure 6d actin.jpg]

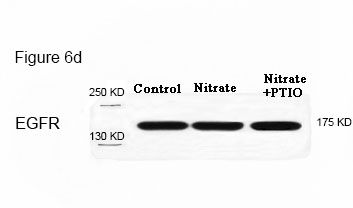

Supplement: Figure 6—source data 4. [file elife-70710-fig6-data4.zip › Figure 6 source data4/Figure 6d EGFR.jpg]

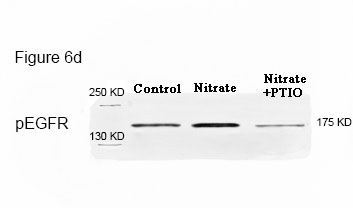

Supplement: Figure 6—source data 4. [file elife-70710-fig6-data4.zip › Figure 6 source data4/Figure 6d pEGFR.jpg]

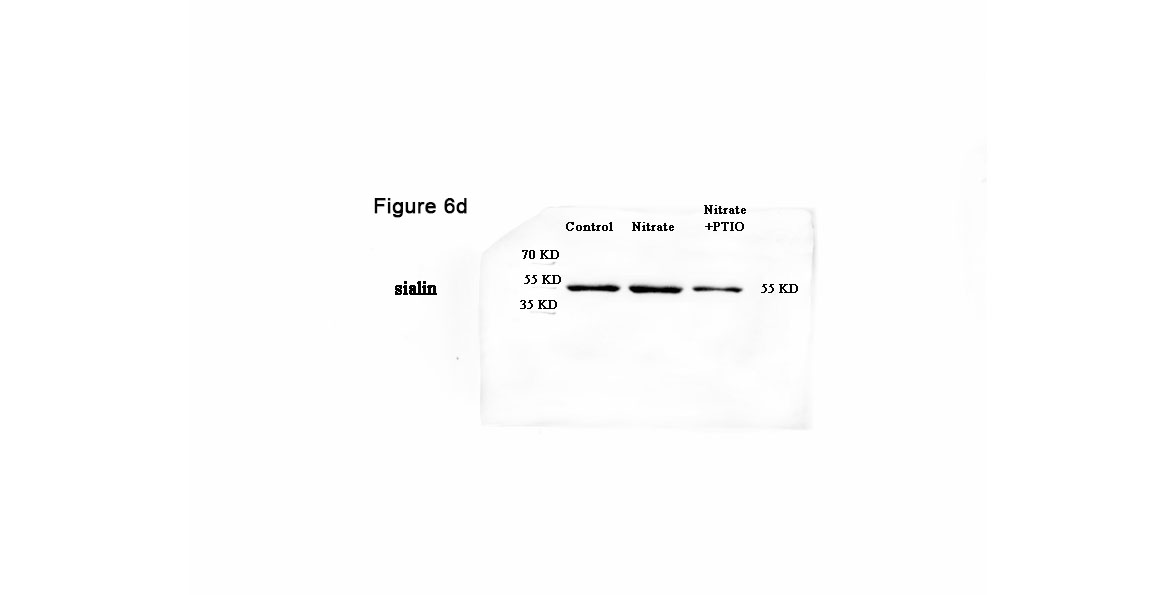

Supplement: Figure 6—source data 4. [file elife-70710-fig6-data4.zip › Figure 6 source data4/Figure 6d sialin.jpg]
