# Supplementary material for: Dietary nitrate supplementation prevents radiotherapy-induced xerostomia
Source: eLife. 2021 Sep 28;10:e70710. doi: 10.7554/eLife.70710 (PMC8563005; doi:10.7554/eLife.70710)
Supplement: Supplementary file 1. [file elife-70710-supp1.docx]

Table 1 Reverse transcription- PCR primer sequences

| Gene | Primer sequence |
| --- | --- |
| Slc17a5 | Forward 5’-AGTGTTATTGTAGCGAGGGACA-3’ |
|  | Reverse 5’-CCTCTGGGAAGCTCGGTCTA-3’ |
| β-actin | Forward 5’-GTCTGCCTTGGTAGTGGATAATG-3’ |
|  | Reverse 5’-TCGAGGACGCCCTATCATGG-3’ |
